# Imaging atherosclerosis with hybrid [^18^F]fluorodeoxyglucose positron emission tomography/computed tomography imaging: What Leonardo da Vinci could not see

**DOI:** 10.1007/s12350-012-9631-9

**Published:** 2012-10-17

**Authors:** Myra S. Cocker, Brian Mc Ardle, J. David Spence, Cheemun Lum, Robert R. Hammond, Deidre C. Ongaro, Matthew A. McDonald, Robert A. deKemp, Jean-Claude Tardif, Rob S. B. Beanlands

**Affiliations:** 1 Molecular Function and Imaging Program, Division of Cardiology, Department of Medicine, University of Ottawa Heart Institute, 40 Ruskin Street, Ottawa, ON K1Y 4W7 Canada; 2 Stroke Prevention & Atherosclerosis Research Centre, Robarts Research Institute, University of Western Ontario, 1400 Western Road, London, ON Canada; 3 Interventional & Diagnostic Neuroradiology, Department of Radiology, The Ottawa Hospital, University of Ottawa, Civic Campus, Diagnostic Imaging, K1Y 4E9 Ottawa, ON Canada; 4 Departments of Pathology and Clinical Neurological Sciences, London Health Sciences Centre and University of Western Ontario, 339 Windermere Road, N6A 5A5 London, ON Canada; 5 Montreal Heart Institute, Université de Montréal, Montreal, Canada

**Keywords:** Positron emission tomography, Computed tomography, [18F]fluorodeoxyglucose, Inflammation, Calcification, Vulnerable plaque

## Abstract

*Prodigious* efforts and landmark discoveries have led toward significant advances in our understanding of atherosclerosis. Despite significant efforts, atherosclerosis continues globally to be a leading cause of mortality and reduced quality of life. With surges in the prevalence of obesity and diabetes, atherosclerosis is expected to have an even more *pronounced* impact upon the global burden of disease. It is *imperative* to develop strategies for the early detection of disease. Positron emission tomography (PET) imaging utilizing [^18^F]fluorodeoxyglucose (FDG) may provide a non-invasive means of characterizing inflammatory activity within atherosclerotic plaque, thus serving as a surrogate biomarker for detecting vulnerable plaque. The aim of this review is to explore the rationale for performing FDG imaging, provide an overview into the mechanism of action, and summarize findings from the early application of FDG PET imaging in the clinical setting to evaluate vascular disease. Alternative imaging biomarkers and approaches are briefly discussed.

Vessels in the elderly restrict the transit of blood through thickening of the tunics.

Leonardo da Vinci, 1452–1519.

## Introduction

Leonardo da Vinci presented perhaps one of the first known descriptions of atherosclerosis. In his collection of notes from post-mortem observations of human anatomy and poorly understood pathology, da Vinci merged what were the distinct disciplines of art and science to describe and depict human development and physiology.^1^ Five-hundred years later, significant advances in technology enable non-invasive visualization of atherosclerosis using multiple imaging modalities, moving beyond anatomical characterization toward directly imaging disease processes and pathophysiology. The current challenge for imaging scientists lies in identifying high-risk atherosclerotic lesions that could lead to coronary or cerebrovascular sequelae prior to adverse vascular events such as myocardial infarction and stroke, and thereby enable personalized therapy while mitigating risk.

Herein, we present a general introduction to advanced non-invasive imaging of atherosclerosis with particular emphasis upon [^18^F]fluorodeoxyglucose (FDG) positron emission tomography (PET). We will discuss the role of FDG PET for the identification of the high-risk “vulnerable plaque” across the arterial tree and its potential for monitoring disease progression and response to therapy.

### Atherosclerosis

Atherosclerosis is the most common underlying pathology responsible for adverse cardiovascular outcomes including angina, myocardial infarction, transient ischemic attacks, and stroke. With an aging population, obesity and diabetes pandemics, cardiovascular disease is projected to become a leading source of global disease burden.^2^-^5^

Atherosclerosis is a chronic disease process that begins with the disruption and inflammation of vascular endothelium, leading to the formation of lipid-rich fatty streaks that may arise as early as infancy. 6 Inflammatory activity within these lesions increases as lipids and macrophages progressively accumulate, resulting in complex remodeling of fibrofatty plaques.^7^,^8^ A typical plaque consists of a central lipid-rich core bound by a fibrous cap. The fibrous cap can suddenly rupture or erode away, exposing the core of the plaque to clotting factors within blood. Activation of these factors results in a cascade that can completely occlude the vessel and induce severe ischemic injury or tissue necrosis.^9^,^10^

Plaque that is thrombosis-prone and likely to progress rapidly, resulting in vessel occlusion is referred to as vulnerable plaque.^11^ Proposed major criteria for defining vulnerable plaque include the presence of active inflammation, particularly increased activated macrophage content.^11^ Evidence suggests that the impending risk to a patient posed by the presence of vulnerable plaque cannot be sufficiently determined by assessing for the anatomic presence of plaque at a specific site or vessel within the arterial tree. Rather, global plaque burden across the entire arterial bed may be a stronger correlate to determine patient risk, thus allowing for risk stratification.^12^ In this regard, non-invasive cardiovascular imaging may be useful as it could visualize disease across the entire arterial bed, and may also be utilized to monitor disease progression or even regression with novel therapies.

### Non-invasive Imaging of Atherosclerosis

#### Contrast-Enhanced Ultrasound

Contrast-enhanced ultrasound takes advantage of portability, bedside imaging and wide availability of carotid ultrasound. Injection of intravascular micro-bubbles permits the visualization of micro- and macro-vasculature, but more importantly, intraplaque neovascularization.^13^ Neovascularization occurs during the early stages of atherosclerosis where undeveloped leaky micro-vessels are formed within the plaque.^14^ The risk associated with these micro-vessels is the development of an intraplaque hemorrhage that potentiates inflammation and contributes toward plaque instability.^14^,^15^ Therefore, neovascularization detected by contrast-enhanced ultrasound may serve as a marker of a high-risk lesion or a vulnerable plaque.^16^ Furthermore, the intima-media is hypoechoic while the adventitia is echogenic, resulting in contrast that can delineate vessel lumen and plaque ulcerations or irregularities on the plaque surface.^17^,^18^ Additionally, intima-media thickness (IMT) can also be measured by contrast-enhanced ultrasound and is a marker of premature atherosclerosis.

## 3-Dimensional Ultrasound

3D ultrasound imaging builds further upon the principles of ultrasound imaging. With 3D ultrasound, it is feasible to visualize carotid plaque and accurately quantify plaque and vessel volume. Plaque volume assessed with 3D ultrasound is a more robust parameter than IMT and thus a highly sensitive means to detect plaque progression, as much as two orders of magnitude better than IMT.^19^-^22^ Plaque grows and extends longitudinally at a faster rate than it thickens. Using 3D ultrasound it is possible to utilize significantly reduced sample sizes to evaluate the impact of an intervention upon plaque progression.^21^ Whether 3D ultrasound can define other high-risk plaque parameters such as ulceration and other aspects of plaque morphology is being evaluated in collaboration with the Canadian Atherosclerosis Imaging Network (clinicaltrials.gov NCT01456403).^23^,^24^

## Computed Tomography (CT)

Computed tomography (CT) enables accurate identification of stenosis within arterial vessels with a high degree of special resolution.^25^ In addition, CT offers the unique ability to characterize calcification within plaque^26^ and stage lesions according to their developmental phase to determine the risk of plaque rupture.^27^ A non-calcified lipid-rich plaque reflects plaque in the early stages of atherosclerosis.^28^ With remodeling, a non-calcified lesion transitions into a mixed plaque that contains calcium deposits and a lipid-rich core.^28^ Progressive accumulation of calcium deposits within plaque leads to the formation of a dense mature calcified plaque. Mixed and non-calcified plaques are at greatest risk for plaque erosion or rupture.^29^ Indeed the presence of spotty calcification in lesions evaluated with multi-slice computed tomography has been associated with acute coronary syndrome.^30^

## Magnetic Resonance Imaging (MRI)

Magnetic resonance imaging (MRI) offers good spatial resolution for the delineation of vascular lumen and offers advantages in demonstrating plaque structure and composition.^31^ MRI can accurately assess mean wall thickness and vessel wall area.^32^ In addition, MRI can lend insight into plaque composition including the lipid-rich core, intraplaque hemorrhage and fibrous cap.^33^-^35^ Compared to CT, MRI does not use ionizing radiation. For these reasons, MRI has been utilized as an endpoint in clinical trials to monitor disease progression and the impact of therapy.^36^ Novel contrast agents that may enable targeted MRI-based molecular imaging of plaque progression are under currently evaluation.^37^-^41^

## Positron Emission Tomography (PET)

**Figure 1:**
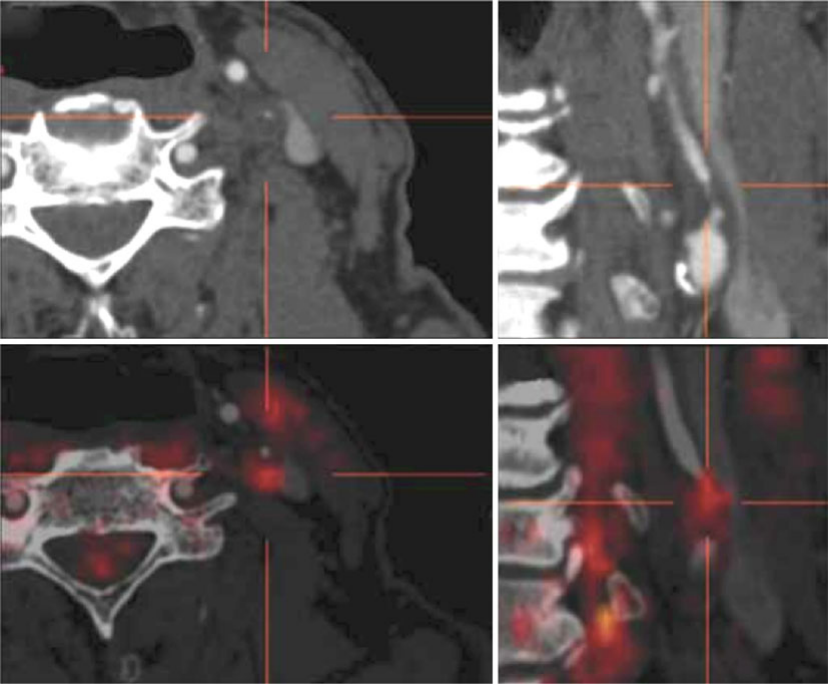
Detection of inflamed plaque in a symptomatic patient a significantly stenotic left internal carotid artery. In transverse and coronal contrast-enhanced CT images (*top row*), there is evidence for significant obliteration of the lumen with little calcification on CT. Hybrid PET/CT images provide evidence for increased [18F]fluorodeoxyglucose at the site of the symptomatic lesion (*bottom row*). 24 (Reproduced with permission of Informa UK, Ltd.)

PET imaging utilizes radiolabeled ligands and tracers that may directly bind to specific targeted molecules or accumulate within specific tissue beds, thereby providing insight into active biologic metabolic processes.^42^ This is an important advantage as it is feasible to probe directly the in vivo expression of molecular and metabolic activity within plaque. In comparison to the anatomic imaging modalities that mainly characterize plaque structure, composition and morphology, PET can evaluate dynamic intraplaque activity such as inflammation, active plaque calcification, and other biologic processes (Figure 1).

## Hybrid PET Imaging

The spatial resolution of clinical PET imaging ranges between 3 and 5 mm.^43^ With such resolution, it is challenging to assess the uptake of radiotracer in small structures such as the carotid and coronary vasculature. To circumvent this limitation, hybrid imaging is performed where PET images are co-registered with either CT or more recently MRI^44^,^45^ (Figure 1). Thus, hybrid imaging may contribute toward greater sensitivity for the anatomical detection of plaque, as well as potentially increased specificity for active disease detection.

## Imaging with [^18^F]FDG PET

**Figure 2:**
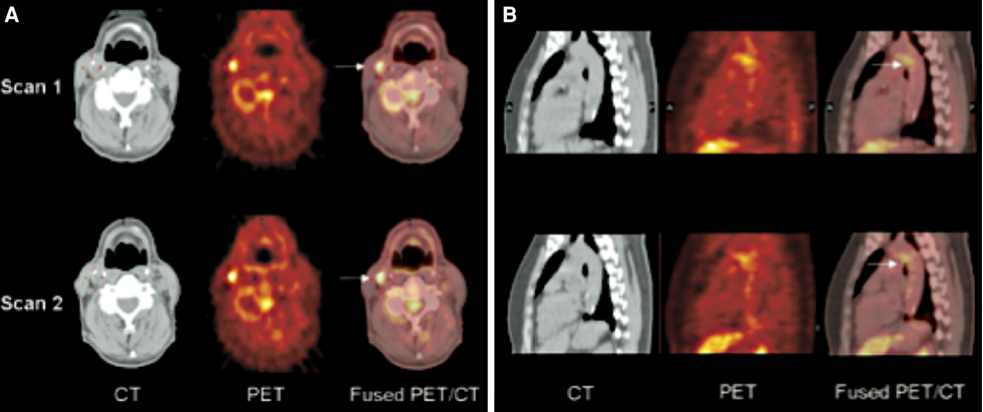
Reproducible carotid and aortic [18F]FDG uptake imaged with hybrid PET/CT over 2 weeks. **A** reflects carotid CT, PET, and hybrid PET/CT images demonstrating reproducible FDG uptake in the right coronary artery (*arrows*). Similarly, **B** is indicative of reproducible FDG uptake at the aortic arch and descending aorta of a patient (*arrows*) (Reprinted from Ref^51^with permission from Elsevier)

“The prime cause of cancer is the replacement of the respiration of oxygen in normal body cells by a fermentation of sugar” (The Prime Cause and Prevention of Cancer, Lecture by Otto Warburg, Annual Meeting of Nobelists, Lindau, Germany, 1966). This pivotal discovery by Nobel Laureate Dr. Otto Heinrich Warburg in 1920s paved the way for future clinical application of [^18^F]FDG imaging. By the early 1980s, FDG was being used to distinguish between benign and malignant lesions, define metabolic activity of cancer cells and myocardium.^46^-^48^ Subsequently, one of the initial applications for imaging vascular inflammation was in the setting of Takayasu Arteritis.^49^ Rudd et al^50^ further expanded the use of FDG for evaluating atherosclerotic plaque within carotid vasculature and subsequently demonstrated that this is a highly reproducible technique (Figure 2).^51^

## The Rationale for [^18^F]FDG PET Imaging of Atherosclerosis

Active inflammation within plaque has been proposed as a major criterion to identify high-risk vulnerable plaque.^12^ Furthermore, the inflammatory burden within ruptured plaque, as reflected by activated macrophages, is significantly increased.^52^ Macrophages potentiate localized inflammatory responses and are pivotal mediators of atherosclerosis. Macrophages themselves have high metabolic rates and require an equally abundant energy supply.^53^ In fact, in comparison to smooth muscle cells, foam cells consume significantly greater amounts of oxygen.^54^ This is partly due to phagocytic activity consisting of oxidative or respiratory bursts that yield superoxides and hydrogen peroxide to degrade engulfed material.^55^

**Figure 3:**
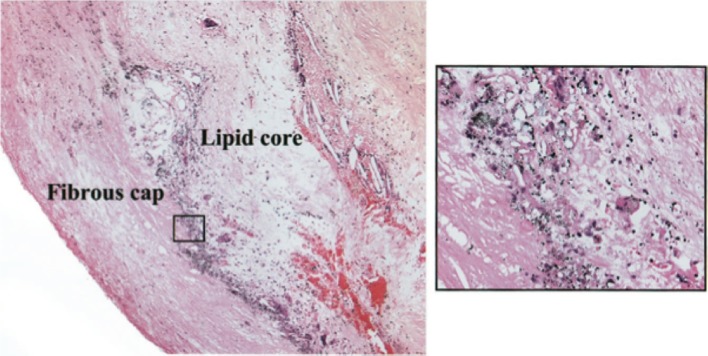
Tritriated deoxyglucose autoradiography of an excised plaque from a symptomatic patient establishes that silver grains accumulate between the lipid core and fibrous cap within macrophages (*inset*) (magnification: ×10 and ×20) (Reprinted from Ref^50^ with permission from Wolters Kluwer Health)

Radiolabelled FDG is a glucose analog that is taken up by active cells to fuel in vivo metabolic processes.^56^ Therefore, FDG uptake may serve as a marker of metabolic activity within a specific tissue. Importantly, given that activated macrophages have high metabolic rates, localized uptake of FDG within plaque may serve as a surrogate marker of an inflamed high-risk lesion. Indeed in patients with a history of transient ischemic attacks arising from a specific carotid artery distribution, as well as significantly stenosed internal carotid artery, FDG was found to localize to macrophage-rich regions^50^ (Figure 3). The question that arises is whether the observed FDG activity is actually reflective of FDG taken up by macrophages, as opposed to surrounding inflammatory cells.

Kubota et al^57^have demonstrated that FDG and 2-deoxy-d-[3H]glucose (3H-DG) uptake is greatest in macrophage-rich regions assessed by macro- and micro-autoradiography in an experimental tumor-induced murine model. Moreover, cellular uptake studies also demonstrate that FDG uptake is almost three-fold greater in macrophages, as compared to tumor cells.^58^ These findings suggest that FDG uptake is a marker of macrophage activity and further research reveals that FDG may also be a marker of early foam cell development.^59^

**Figure 4:**
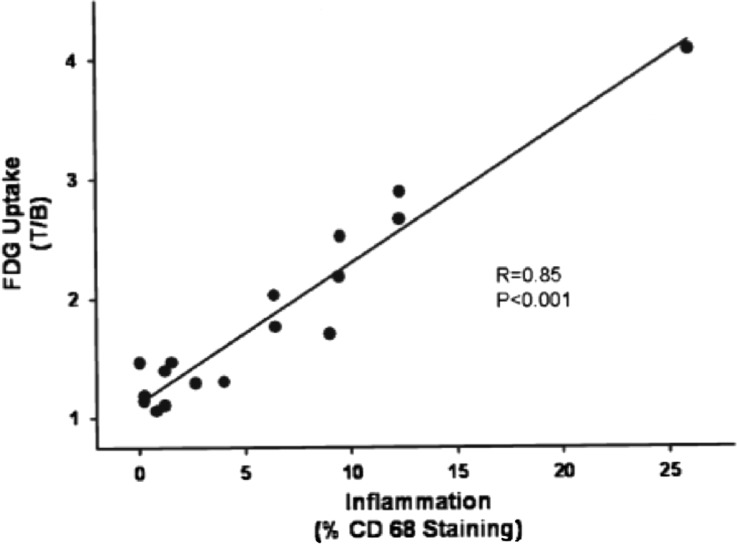
Mean within-patient [18F]fluorodeoxyglucose uptake (expressed as a target-to-background ratio) is significantly correlated with inflammation (*r* = 0.85; *P* < .001). Inflammation was defined as the percent of CD68 macrophage staining with immunohistology (Reprinted from Ref^60^ with permission from Elsevier)

Finally, pioneering work by Rudd et al,^50^ Tawakol et al,^60^ and others^61^-^66^ supports an association between FDG uptake quantified in human carotid plaque specimens with macrophage-specific CD68 immunohistology staining (Figure 4).

## Insights into Atherosclerotic Disease Across Vascular Beds Derived from [^18^F]FDG PET

### Carotid Vasculature

The relationship between carotid plaque composition and FDG uptake has been evaluated in several studies.^67^ In terms of calcification, an inverse relationship between calcium content within carotid plaque and FDG uptake is noted.^61^ Lipid-rich necrotic plaque has been shown to have greater FDG uptake than collagenous or calcified plaque.^68^ Additionally, FDG uptake has also been related to high-risk morphological features of carotid plaque such as positive remodeling, low attenuation profile (suggestive of a lipid-rich core), and luminal irregularity (marker of plaque ulceration), as characterized with hybrid PET/CT imaging,^62^ inferring that FDG uptake may identify patients at higher risk of vascular events. Indeed FDG uptake within carotid vasculature has been related to cerebral micro-embolism^69^ and risk of stroke.^70^

FDG uptake in carotid vasculature has been shown to correlate with serum levels of C-reactive protein, a marker of systemic inflammation.^67^,^71^ This supports the concept of defining a “vulnerable patient” whereby the presence of inflamed plaque within one node of the arterial tree may increase the likelihood for the presence of a vulnerable lesions within other vascular beds.^71^ Furthermore, other pre-existing co-morbidities may contribute toward the burden of risk in certain patient populations. Patients presenting with impaired glucose tolerance and type-2 diabetes mellitus have increased carotid FDG uptake that correlates with Framingham risk score.^72^ Likewise, patients presenting with metabolic syndrome or with features suggestive of metabolic syndrome also have increased carotid FDG uptake.^73^,^74^

There is also evidence that suggests that symptomatic carotid lesions tend to have greater FDG uptake when compared to asymptomatic lesions, although the extent of vascular stenosis is an important determinant.^50^ In general, while the degree of vascular stenosis evaluated with angiography is related to FDG uptake, 25% of non-stenotic lesions detected in a vascular territory compatible with patient presentation using high-resolution MRI angiography has significantly inflamed plaques that can be imaged with FDG.^75^ Therefore, FDG imaging of atherosclerotic lesions may be of incremental benefit when performed in conjunction with angiography (e.g., CTA or MRA) to identify culprit lesions at high risk of rupture.^75^

### Large Arterial Vasculature

Possibly, the first clinical experience of imaging FDG uptake in large arterial vasculature was reported by Yun et al.^76^ From a cohort of 156 patients referred for various clinical indications, Yun et al^77^ reported that 51% of subjects had evidence for FDG uptake at the level of the abdominal aorta, 51% at the iliac, and 63% at proximal femoral arteries. Along all vascular beds, age and hypercholesterolemia were correlated with the degree of FDG uptake.^77^ Of further interest, a variation existed among the atherosclerotic risk factors associated with FDG uptake across vascular beds.^77^ These risk factors included age and hypercholesterolemia (abdominal aorta and iliac arteries), hypertension (iliac arteries), and diabetes (femoral arteries).^77^ These findings suggest that each atherogenic risk factor may have non-uniform potency in contributing to disease progression in different vascular beds. In addition to inflammation, aging has been associated with increased aortic wall and calcification volume, as well as metabolically active inflamed lesions^78^-^80^ Female patients, patients with cardiovascular disease and those with cardiovascular risk factors have also been noted to present with increased FDG uptake or highly inflamed plaques, while diabetic patients may have more pronounced aortic calcification.^81^

### Coronary Vasculature

Imaging of the coronary vasculature has proven more challenging due to the small size of these vessels. Confounders such as cardiac motion, myocardial FDG uptake and the spatial resolution of PET have impeded progress in imaging FDG uptake in coronary vessels. Despite such challenges, Dunphy et al^82^ presented what may be the first clinical experience of imaging coronary vasculature using FDG. They evaluated calcification and FDG uptake within the coronary arteries of 78 patients referred for oncology imaging^82^ and found that there was a significant correlation between coronary FDG uptake and abnormal myocardial perfusion in 32 patients.^82^ FDG uptake was also associated with cardiac risk factors, although no cardiac events were reported during a follow-up period of 7 months.^82^ However, 34 patients were excluded from the analysis due to severe motion artifacts. Similarly, in a larger cohort assessed by Saam et al,^83^ FDG uptake at the left anterior descending artery would only be assessed 55% of patients. Nonetheless, uptake was related to hypertension, coronary heart disease, body mass index, calcified plaque burden, and pericardial fat volume.

In order to suppress myocardial FDG uptake, Wykrzykowska et al^84^ instructed patients to consume a low-carbohydrate, high-fat meal the night prior to imaging and drink a vegetable oil-based drink on the morning of the imaging study. In 32 patients with a history of treated malignancy and who underwent both FDG PET/CT and cardiac catherization, good cardiac (muscle) FDG suppression was achieved in 63% of patients.^84^ Coronary FDG uptake was identified in 15 patients, and when compared to angiography results, there was a trend toward an association between anatomic disease and metabolic FDG uptake.^84^ Furthermore, FDG uptake in the left main coronary artery has been shown to be higher in patients presenting with acute coronary syndrome when compared to those with stable angina.^85^

### Multi-vascular Disease

The interrelationship of atherosclerotic disease across arterial beds has also been evaluated using FDG imaging. Studies suggest that among different vascular beds, there is evidence for variable FDG uptake,^82^,^86^ while there is a positive relationship between uptake in adjacent territories and along paired left and right arterial beds.^87^ Multi-vascular evaluation of disease has been proposed, given that high levels of FDG uptake across major vascular beds including the aorta, iliac, and carotid arteries has been shown to be predictive of future cardiovascular events.^88^

In terms of identifying patients at greatest risk, older patients presenting with more cardiovascular risk factors tend to have more inflamed active and calcified inactive plaques.^89^ Although quantification of FDG uptake is a highly reproducible measure, it does vary over time suggesting that inflammation may be a transient feature of atherosclerosis which waxes and wanes as disease progresses.^51^,^90^-^92^

## The Emergence of [^18^F]FDG in Clinical Trials

**Figure 5:**
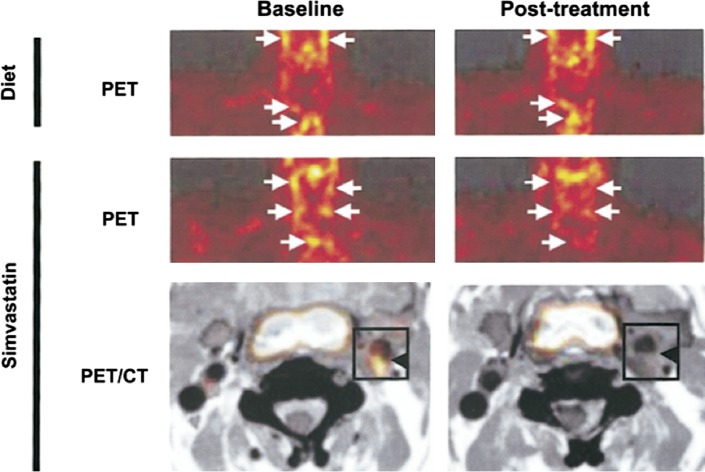
Reduced [18F]FDG following simvastatin therapy. Representative images of a patient on dietary management alone (*top row*). Three-months of dietary management alone had no impact upon FDG uptake in aortic and carotid vasculature (*arrows*). However, FDG uptake is visibly reduced in the carotid arteries and aortic arch following 3 months of therapy with simvastatin (*middle row*). Hybrid FDG PET/CT images demonstrate that following 3 months of therapy with simvastatin, there is no evidence for visible FDG uptake (*bottom row*) (Reprinted from Ref^94^with permission Elsevier)

Serial FDG PET scanning has the potential to evaluate changes in inflammatory activity. Lifestyle modification induces a detectable reduction in FDG uptake within aortic and iliac vasculature.^93^ Likewise, reduced FDG uptake in the aorta and carotid vessels has been observed in association with Simvastatin lipid-lowering therapy^94^ (Figure 5). Furthermore, in patients randomized to low- and high-dose Atorvastatin therapy and followed for 6 months, high-dose therapy was associated with significantly reduced FDG uptake at the femoral artery and aorta.^95^ A similar reduction in inflammation has also been observed following treatment with Atorvastatin for 12 weeks.^96^

More recently, 130 patients were randomized to either dalcetrapib (a novel HDL raising drug) or placebo to determine whether dalcetrapib modulates plaque progression and inflammation.^36^ Treated patients had reduced FDG uptake at the most diseased segments of carotid artery.^36^ Upon dividing patients into three tertiles according to total change in vessel area at 24 months, patients in the two lowest tertiles had reduced vascular inflammation observed after 6 months (*P* = .01).^36^ Thus, FDG PET may be a sensitive and specific marker to non-invasively monitor disease progression and response to therapy. Ongoing trials such as the Canadian Atherosclerosis Imaging Network (CAIN) will contribute toward validating and establishing FDG PET against advanced immunohistology, as well as develop other imaging-derived biomarkers using a multimodality approach to characterize aspects of plaque biology, disease burden, and identifying high-risk lesions.

## Limitations of PET/CT Imaging

Given that FDG imaging of vasculature is a relatively new area of science and investigation, it suffers from several limitations. Although FDG uptake is related to macrophage expression, direct evidence demonstrating that FDG is taken up directly into macrophage cells is still lacking. This may be achievable in the future with higher resolution imaging and amplified immunohistology. Imaging coronary vasculature with FDG continues to be hampered by myocardial motion and myocardial FDG uptake. Acquiring respiratory- and cardiac-gated images may reduce the impact of coronary vessel motion. Furthermore, as described, patient preparation and diets may lower myocardial FDG uptake.

Serial imaging is required to accurately stage atherosclerosis as it matures, waxes, and wanes. PET and CT imaging requires exposure to radiation which currently precludes the feasibility of frequent serial imaging. Algorithms to reduce patient radiation exposure are under development and will eventually contribute toward enabling serial imaging. Hybrid PET/MRI and PET/CT imaging also require accurate image co-registration between both modalities, especially when evaluating small structures such as the internal carotid vasculature. Slight patient motion between image acquisitions can result in misalignment that can bias attenuation correction, precluding accurate co-localization, and image interpretation. The use of neck-braces and hybrid scanning systems can significantly reduce this.

## Emerging PET/CT Imaging Probes for Human Atherosclerosis

With the limitations of FDG and the complexity of atherosclerotic process, other surrogate makers of inflammation have also been used in human including: [11C]PK11195, [11C]choline and 68Ga-[1,4,7,10-tetraazacyclododecane-*N*,*N*′,*N*″,*N*9′′′-tetraacetic acid]-D-Phe1, Tyr3-octreotate (DOTATATE) (Table 1). [11C]PK11195 is a selective ligand of a translocator protein that is highly expressed by macrophages.^97^-^99^ Similarly, [68Ga]DOTATATE binds to somatostatin receptors subtype 2 that are expressed by macrophages.^100^ The uptake of [11C]PK11195 and [68Ga]DOTATATE are considered to be indicative of macrophage density within plaque. [11C]choline differs in that it is taken up by inflammatory cells—primarily macrophages, following which it undergoes phosphorylation and is metabolized into forming phosphatidylcholine that is eventually incorporated into the cellular membrane.^101^ Other tracers such as Annexin-V that are currently being utilized to detect apoptosis may be more specific markers of phagocytosing macrophages are being evaluated as single-photon emission computed tomography radiotracers but can also be labeled with 18F.^102^-^104^

**Table 1:** PET/CT radiotracers that have been applied in the clinical setting for characterizing plaque

PET radiotracer	Mechanism of action	Uptake suggestive of	Arteries evaluated	Validation in human atherosclerotic plaque
[^18^F]fluorodeoxyglucose (FDG)^60^	Uptake by metabolically active cells	Macrophage density	Carotid, aorta, coronary, iliac, femoral	Immunohistochemistry and autoradiography
^11^C-PK11195^97^-^99^	Selective ligand of the translocator protein (TSPO, 18 kDa), formerly known as peripheral benzodiazepine receptor	Macrophage density	Carotid, aorta (vasculitis )	Immunohistochemistry and autoradiography
^11^C-choline^101^	Choline enters the cell via specific transport mechanisms, is phosphorylated by choline kinase, metabolized to phosphatidylcholine is incorporated into the cell membrane	Macrophage density, inflammatory infiltrates	Carotid, aorta	No
^68^Ga-[1,4,7,10-tetraazacyclododecane-*N*,*N*′,*N*″,*N*9′′′-tetraaceticacid]-d-Phe1, Tyr3-octreotate (DOTATATE)^100^	Binds to somatostatin receptors of subtype 2 (SSTR2)	Macrophage density	Coronary	No
^11^C-Acetate^116^	Fatty acid synthesis in lesions requires acetyl-coenzyme-A, which is produced from acetate	Fatty acid synthesis	Carotid, aorta, iliac	No
[^18^F]Sodium fluoride^117^-^119^	Binds to hydroxyapatite molecules by replacing hydroxyl groups	Calcification	Carotid, aorta, iliac, femoral, coronary	No

**Figure 6:**
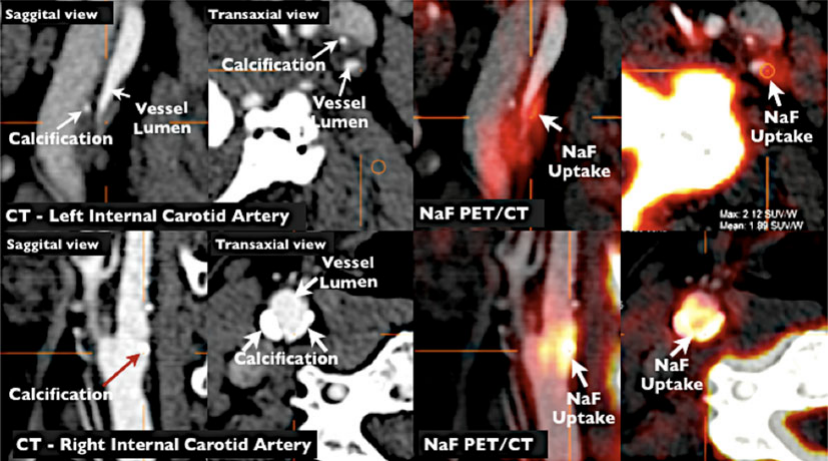
NaF PET/CT imaging of left and right internal carotid arteries of active calcification in a 72-year-old symptomatic patient evaluated at the University of Ottawa Heart Institute. *Upper row* evidence of NaF uptake with a small foci of calcification on CT in the left internal carotid symptomatic culprit vessel. There is a mismatch between the region of NaF uptake and calcification on CT. *Lower row* Evidence of calcium nodules with matched NaF uptake at the right internal carotid artery

In addition to inflammation, calcification of plaque may also contribute toward potentiating plaque vulnerability.^105^-^107^ Hydroxyapatite is expressed in regions with active calcium deposition. [^18^F]sodium fluoride (NaF) binds to hydroxyapatite molecules by replacing hydroxyl groups, and could, therefore, serve as a surrogate marker of active calcification within plaque^108^ (Figure 6). Early findings suggest that NaF uptake imaged with PET may identify regions of active calcium deposition.^109^ Recently, NaF has been applied in coronary vasculature where compared to FDG, there is very little competing radiotracer uptake by myocardium resulting in high target-to-background signal.^109^

These tracers are still very early in development and application. Further studies are required to establish and validate them prior to wider application in clinical research and potential future clinical application.

## Integration of PET/CT Imaging Biomarkers to Understand Plaque Progression

Utilizing PET/CT imaging biomarkers, it may be possible to non-invasively stage lesions and assess different aspects of plaque progression. Among a cohort of 45 oncology patients, plaque was characterized for inflammation (FDG uptake), active mineral deposition (NaF uptake) and calcification (CT).^109^ Of 105 lesions that had NaF uptake, 81 (77.1%) had evidence of calcification on CT, while 18 (14.5%) had evidence of FDG uptake.^109^ Therefore, one could hypothesize that imaging macrophages with FDG, active calcification imaging with NaF and calcium deposition with computed tomography could be markers of independent processes, and that these markers could be utilized to stage atheroma formation, disease progression and potentially plaque and patient vulnerability.

**Figure 7:**
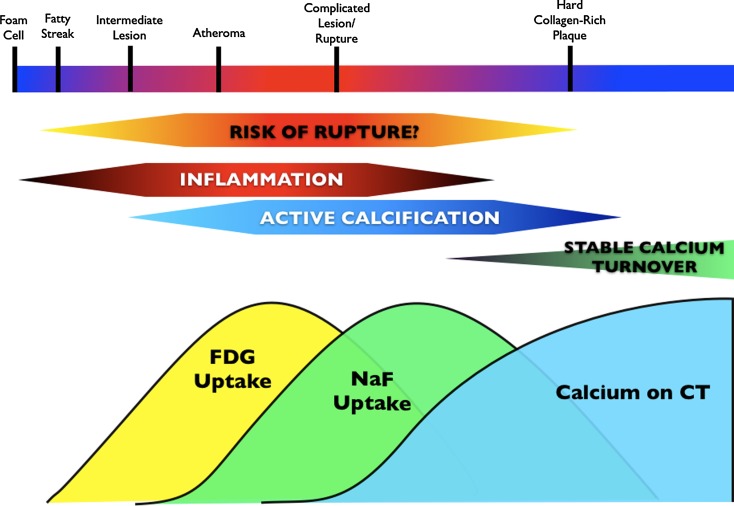
A proposed schematic staging inflammatory and calcification activity within atherosclerotic lesions with FDG and NaF as imaging biomarkers. During early stages of atherosclerosis, inflammation is the predominant mechanism active within plaque. During these stages, [18F]FDG may be taken up by the lesion. As inflammation peaks, the risk of plaque rupture may increase. Inflammation also contributes toward initiating calcium metabolism within lesions that results in the formation of early calcium deposits. This would be reflected by uptake of both FDG and hydroxyapatite-specific [18F]sodium fluoride (NaF). Once the density of calcium deposits exceeds a certain threshold, it becomes visible with CT. During active calcification, plaque may still be vulnerable. Eventually, the calcification and mineralization processes exceed the inflammatory activity present within plaque, which might be demarcated by only NaF uptake (in the absence of FDG), as well as calcium deposits on CT. Ongoing calcification eventually leads to forming an end-stage stable atheroma that is densely calcified with only evidence for calcium on CT. Model of plaque progression (*top bar*) is adapted from Koenig and Khuseyinova^115^

One may speculate that in the early stages of advanced atherosclerosis, only FDG uptake would be detected as inflammation is predominant process present (Figure 7). Active calcification progressively initiates given that the inflammatory cascade contributes to calcium deposition. As inflammation peaks, early calcium deposits would also be present. From imaging, this phase of atheroma progression would be reflected by uptake of both FDG and NaF. Once the density of calcium deposits exceeds a certain threshold, these would also be visible on CT—possibly as early speckled calcification (a presumed marker of risk on CT angiography studies).^30^ Eventually, calcification and mineralization processes would exceed the inflammatory activity present within plaque. At this stage, there would be evidence of NaF uptake in the absence of FDG as well as calcium deposits on CT. Ongoing calcification may finally lead to development of an end-stage longstanding atheroma that is densely calcified with very little active calcium turnover. At this final stage, there would only be evidence for calcium on CT.

## Conclusion

Considerable advancement has occurred in imaging atherosclerosis, with FDG PET/CT at the forefront for characterizing actively inflamed plaque. FDG uptake within plaque has transitioned from an incidental finding to a potential biomarker of vulnerable plaque that has even been implemented to evaluate the efficacy of vascular risk reduction therapy. Despite the proliferation of imaging research, significant progress is yet to be achieved in fully understanding the molecular biology of plaque progression and rupture. Recent pre-clinical developments may help in understanding how FDG and other novel tracers track inflammation in plaque.^42^,^110^,^111^

While FDG imaging has enhanced our understanding of the inflammatory processes that underlie atherosclerosis, further studies are needed to validate FDG uptake. It also remains to be determined whether FDG uptake within plaque could be a specific marker of activated, polarized M1 (pro-inflammatory) or M2 (anti-inflammatory) macrophages,^112^ as validated by advanced immunohistology. Furthermore, whether FDG uptake is truly predictive of future cardiovascular events and outcomes remains to be determined. Also, while reductions in FDG uptake have been observed following therapies that modify vascular risk, to date, a reduction in subsequent downstream vascular events has only been demonstrated in small observational studies. Prospective trials will further yield insight into the role of FDG to detect inflamed carotid plaque, as validated by advanced immunohistochemistry in patients with high-risk carotid artery disease^36^,^113^ and its role in imaging other vascular beds as in the BIOIMAGE study.^114^ Furthermore, although FDG has advantages in light of its widespread availability and is currently the PET/CT imaging biomarker of choice for large trials evaluating plaque inflammation and progression, whether it will be the optimal PET/CT plaque imaging biomarker remains to determined.

While we have moved far beyond da Vinci’s initial gross observations 500 years ago to visualizing inherent processes within plaque, we still need to bridge the gap between translating imaging of atherosclerosis to accurately predicting life-threatening vascular events. At that point, we may begin to realize imaging-guided personalized care for patients with atherosclerosis.
